# Antimicrobial activity of the *Lacticaseibacillus rhamnosus* CRL 2244 and its impact on the phenotypic and transcriptional responses in carbapenem resistant *Acinetobacter baumannii*

**DOI:** 10.1038/s41598-023-41334-8

**Published:** 2023-08-31

**Authors:** Cecilia Rodriguez, Dema Ramlaoui, Nardin Georgeos, Briea Gasca, Camila Leal, Tomás Subils, Marisel R. Tuttobene, Rodrigo Sieira, Nicholas T. Salzameda, Robert A. Bonomo, Raúl Raya, María Soledad Ramirez

**Affiliations:** 1 Centro de Referencia Para Lactobacilos (CERELA), CONICET, San Miguel de Tucumán, Tucumán Argentina; 2 Center for Applied Biotechnology Studies, Department of Biological Science, College of Natural Sciences and Mathematics, California State University Fullerton (CSUF), 800 N State College Blvd, Fullerton, CA 92831 USA; 3 Instituto de Procesos Biotecnológicos y Químicos de Rosario (IPROBYQ, CONICET-UNR), Rosario, Argentina; 4 Instituto de Biología Molecular y Celular de Rosario (IBR, CONICET-UNR), Rosario, Argentina; 5 Fundación Instituto Leloir-IIBBA CONICET, Buenos Aires, Argentina; 6 Department of Chemistry and Biochemistry, College of Natural Science and Mathematics, CSUF, Fullerton, USA; 7 Research Service and GRECC, Department of Veterans Affairs Medical Center, Louis Stokes Cleveland, Cleveland, OH 44106 USA; 8 Departments of Medicine, Pharmacology, Molecular Biology and Microbiology, Biochemistry, Proteomics and Bioinformatics, Case Western Reserve University School of Medicine, Cleveland, OH 44106 USA; 9 CWRU-Cleveland VAMC Center for Antimicrobial Resistance and Epidemiology (Case VA CARES), Cleveland, OH 44106 USA

**Keywords:** Antimicrobials, Pathogens, Transcriptomics

## Abstract

Carbapenem-resistant *Acinetobacter baumannii* (CRAB) is a recognized nosocomial pathogen with limited antibiotic treatment options. Lactic acid bacteria (LAB) constitute a promising therapeutic alternative. Here we studied the antibacterial properties of a collection of LAB strains using phenotypic and transcriptomic analysis against *A. baumannii* clinical strains. One strain, *Lacticaseibacillus rhamnosus* CRL 2244, demonstrated a potent inhibitory capacity on *A. baumannii* with a significant killing activity. Scanning electron microscopy images showed changes in the morphology of *A. baumannii* with an increased formation of outer membrane vesicles. Significant changes in the expression levels of a wide variety of genes were also observed. Interestingly, most of the modified genes were involved in a metabolic pathway known to be associated with the survival of *A. baumannii*. The *paa* operon, Hut system, and fatty acid degradation were some of the pathways that were induced. The analysis reveals the impact of *Lcb. rhamnosus* CRL 2244 on *A. baumannii* response, resulting in bacterial stress and subsequent cell death. These findings highlight the antibacterial properties of *Lcb. rhamnosus* CRL 2244 and its potential as an alternative or complementary strategy for treating infections. Further exploration and development of LAB as a treatment option could provide valuable alternatives for combating CRAB infections.

## Introduction

Carbapenem-resistant *Acinetobacter baumannii* (CRAB) has gained notoriety in recent years due to its rapid nosocomial emergence and global spread. CRAB causes severe infections in vulnerable patients and is also known to colonize the rectum of patients and workers associated with intensive care units^1–3^. The circulating CRAB strains possess extreme antibiotic resistance (XDR) and in some cases pan-drug resistance (PDR)^4^, which severely complicates therapy with currently available antibiotics. In the last decade, and despite numerous efforts to find therapeutic alternatives, the production of new drugs for the treatment of infections caused by CRAB has been scarce.

Lactic acid bacteria (LAB) constitute a promising therapeutic alternative due to the demonstrated ability of certain LAB strains to inhibit ESKAPE group pathogens (*Staphylococcus aureus*, *Escherichia coli*, *Klebsiella pneumoniae*, *Acinetobacter baumannii*, *Pseudomonas aeruginosa*, and *Enterococcus faecalis*)^5–9^. LAB are Gram-positive microorganisms considered safe for inclusion in food (GRAS) and are widely used in the production of various fermented foods, where they contribute taste and texture of the final product^10,11^. LAB are widely distributed in nature and many of them are found as part of the gut microbiota of humans and animals. Strains of different LAB species are used as probiotic supplements for their beneficial properties for human or animal health. These benefits range from improving intestinal health and immune response^12^, to preventing acute and antibiotic-associated diarrhea^13^, and chronic gastritis^14,15^, among others. The antimicrobial effect of LAB against pathogens constitutes an important property in the selection of potential probiotics for the maintenance of intestinal microbial balance and as a substitute for synthetic antibiotics^16^. Probiotic LAB are antagonistic to pathogens and inhibit the growth of these bacteria by (i) producing antimicrobial substances or bioactive compounds^7–9,17^; (ii) by occupying their niches and/or displacing them (including in the prevention and/or elimination of biofilms)^5^; (iii) by promoting gut maturation and integrity, and increasing the non-immune-dependent barrier effect; or by directly activating lymphoid cells, partly mediated by gut-associated lymphoid tissue (GALT system), and (iv) modulating local as well as systemic immune responses^6,18,19^.

The antimicrobial activity of certain LAB and/or their extracellular products against *A. baumannii* have been described^20^. In a murine model of respiratory infection, demonstrated that *Streptococcus constellatus*, frequently isolated from the oral cavity, enhances the immune response of mice by promoting the proliferation of cytotoxic lymphocytes that eliminate *A. baumannii*. Stanbro et al.^19^ demonstrated that topical application of certain products of *Lcb. acidophilus* ATCC 4356 and *L. reuteri* ATCC 23272 were effective in resolving wounds caused by *A. baumannii*. Furthermore, in vitro studies demonstrated the antagonistic activity of different LAB strains such as *L. animalis* LMEM6, *L. plantarum* LMEM7, *L. acidophilus* LMEM8, *Lcb. rhamnosus* LMEM9*, Lcb. casei* Shirota, *L. plantarum* LJ1R3, and *L. gasseri* LBM220 against clinical isolates of *A. baumannii* MDR^5,8,21,22^. While there is evidence that LAB exert an antagonistic effect against *A. baumannii*, the specific response of *A. baumannii* and the impact of LAB on its survival, virulence, and persistence have not been thoroughly examined. The aim of this study is to address the knowledge gap regarding the response of *A. baumannii* to the antagonistic effect of LAB and to investigate how the presence of LAB affects the behavior of *A. baumannii* providing insights that can contribute to strategies for the control of nosocomial infections. Herein, we will assess various aspects of *A. baumannii'*s behavior, including its growth rate, biofilm formation, antibiotic susceptibility, and expression of virulence associated genes.

## Results

### Inhibitory effect of ten different LAB on *A. baumannii* clinical strains

**Figure 1:**
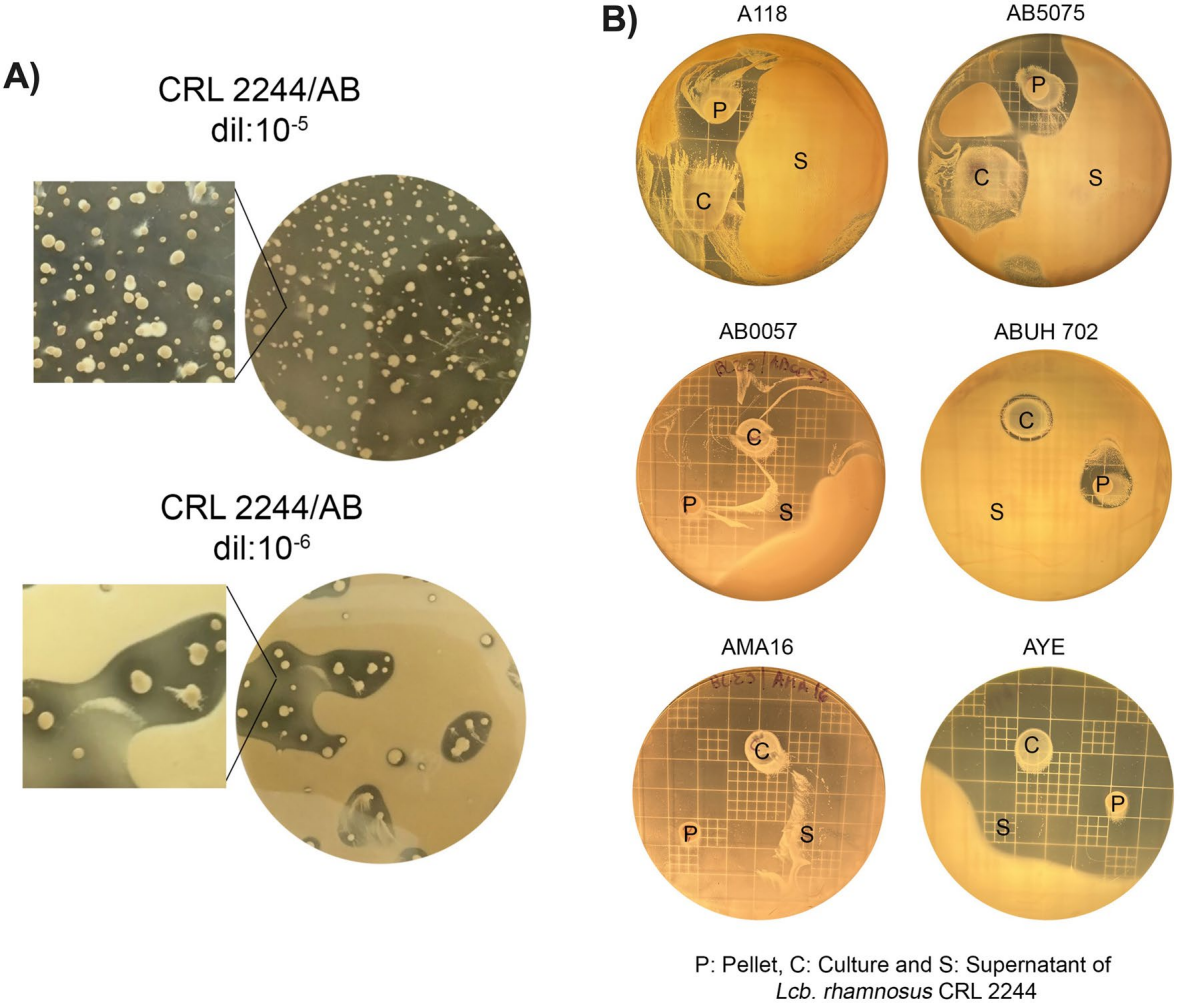
Inhibitory effect of *Lcb. rhamnosus* CRL 2244 on *A. baumannii* clinical strains. (**A**) Inhibitory activity on *A. baumannii* A118 (AB). The plate of dilution 10^–5^ shows the fully inhibited AB lawn, while the plate of dilution 10^–6^ shows the inhibition halos around the CRL 2244 colonies. (**B**) Antimicrobial activity of culture (**C**), pellet (P) and cell supernatant (S) spot of *Lcb. rhamnosus* CRL 2244 on carbapenem-resistant *A. baumannii* strains at 16 h incubation.

We initially evaluated the inhibitory activity of ten different LAB species on the antibiotic-susceptible representative strain *A. baumannii* A118 by agar overlay testing the overnight culture, the collect pellet, and the supernatant of the LABs. In the solid medium bacterial interaction assays, most of the tested strains showed low or absent inhibitory capacity on A118 (Table 1). However, strong inhibition activity defined as “high R value” was observed around the culture and pellet of *Lcb. rhamnosus* CRL 2244 (7 and 7.5, respectively) (Table 1). The antagonistic effect represented as arbitrary units/ml reached 1600 and 1650 for culture and pellet of *Lcb. rhamnosus* CRL 2244, respectively (Table 1) Particularly, the same “strong” inhibition halos on A118 were also observed when serial dilutions of an overnight culture of CRL 2244 were seeded on a 10^8^ CFU lawn of the indicator model strain A118 (Fig. 1A).

**Table 1:** Soft agar overlay assay.

Strains	IHD (mm)	SD (mm)	R	AU/ml
*L. curvatus* CRL705	C = 16	C = 10	3 L	800
	P = 18	P = 8	5 L	900
	S = 0	S = 0	WI	0
*L. mucosae* CRL573	C = 16	C = 11	2.5 L	800
	P = 16	P = 9	3.5 L	800
	S = 0	S = 0	WI	0
*L. acidophilus* CRL641	C = 0	C = 11	WI	0
	P = 15	P = 9	3 L	750
	S = 0	S = 0	WI	0
*F. tropaeoli*s CRL2034	C = 12	C = 10	WI	600
	P = 12	P = 9	WI	600
	S = 0	S = 0	WI	0
*L. reuteri* CRL1101	C = 7	C = 6	WI	350
	P = 16	P = 9	3.5 L	800
	S = 0	S = 0	WI	0
*C. farciminis* CRL748	C = 0	C = 8	WI	0
	P = 15	P = 8	3.5 L	750
	S = 0	S = 8	WI	0
*Lcb. rhamnosus* ATCC53103	C = 0	C = 8	WI	0
	P = 12	P = 8	WI	600
	S = 0	S = 8	WI	0
*Lcb. rhamnosus* CRL75	C = 15	C = 11	2 L	750
	P = 15	P = 8	3.5 H	750
	S = 6	S = 6	WI	300
*Lcb. rhamnosus* Principia	C = 10	C = 8	WI	500
	P = 14	P = 8	3 L	700
	S = 0	S = 8	WI	0
*Lcb. rhamnosus* CRL 2244	C = 32	C = 9 (18)	7 H	1600
	P = 33	P = 8 (18)	7.5 H	1650
	S = 0	S = 6	WI	0

Antimicrobial activity of culture (C), pellet (P) and supernatant (S) spot of LAB on *A. baumannii* A118 at 16 h incubation. Inhibition halos diameter (IHD) were measured, and inhibitory capacity was interpreted > 20 mm = High (H), 20–10 mm = Intermediate (I) and < 10 mm = Low (L). The width of the clear zone (R) is the difference between IHD and spot diameter divided by two (SD), inhibitory capacity was interpreted as R > 6 mm = High (H), 5–2 mm = Low (L), < 2 mm = Without Inhibition (WI). The arbitrary units per ml (AU/ml) was calculated as the IHD × 1000 divided by the μl seed (Halder et al.^5^). The average of three independent assays is represented on the table.

Therefore, *Lcb. rhamnosus* CRL 2244 was selected to evaluate its antimicrobial activity by agar overlay against CRAB strains from different clonal complexes, harboring different types of carbapenemases and with extreme antibiotic resistance (XDR). In addition, a model probiotic strain commonly used to treat or prevent diarrhea, *Lcb. rhamnosus* ATCC 53103 (GG), was included. CRL 2244, in the assayed conditions, inhibited strains AB5075 (hypervirulent strain), AMA16, AB0057, ABUH702 and AYE, with "strong" inhibition halo diameters (DHI > 20 mm); however, inhibition of strain ABUH702 was weak (DHI < 10 mm) (Fig. 1B). The strain ATCC 53103, in the assayed conditions, did not show antimicrobial activity on any of the CRAB strains evaluated (data not shown).

### *Lcb. rhamnosus* CRL 2244 possessed a potent killing activity against carbapenem-susceptible and CRAB strains

To evaluate the killing activity of *Lcb. rhamnosus* CRL 2244, we assessed the viability of AB5075 and A118 24 h after co-incubation of cells with CRL 2244. Both cells in ratio 1:1 and in stationary phase (without the addition of fresh medium) were used. Total cell death was observed in both cases with a survival rate less than 1 × 10^–9^ and less than 1 × 10^–8^ for AB5075 and A118, respectively.

**Figure 2:**
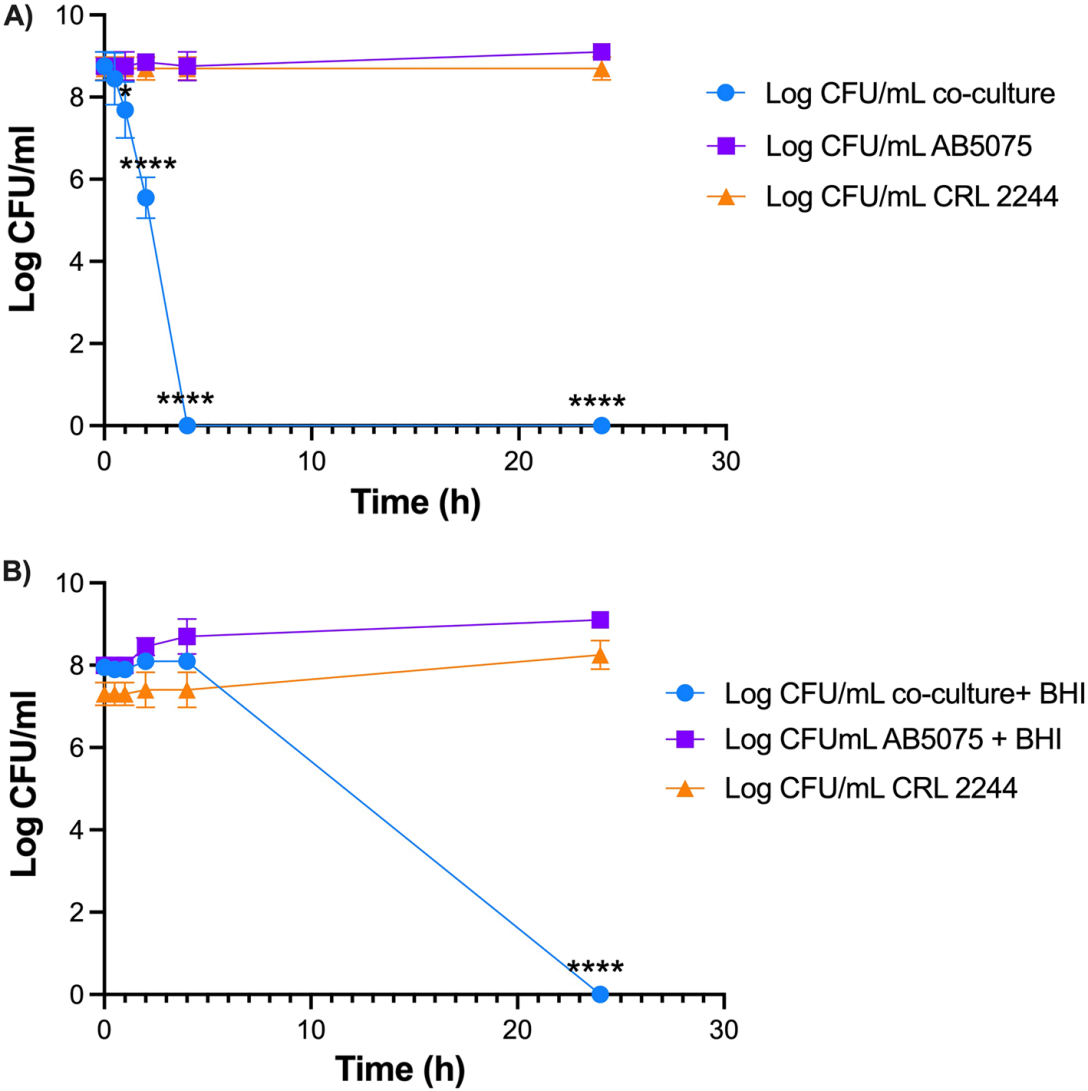
Killing activity of *Lcb. rhamnosus* CRL 2244 on *A. baumannii* AB5075. (**A**) Cells grew overnight and combined in a 1:1 ratio (**B**) Overnight cultures of both cells in 1:1 ratio with addition of fresh medium. Both conditions were incubated at 37 °C for 24 h and CFU/mL were determined at different incubation times for a period of 24 h. All assays were carried out in duplicate. Statistical significance (*P* < 0.05) was determined by two-way ANOVA followed by Tukey’s multiple comparison test. Significance of co-culture vs CRL2244 and co-culture vs AB5075 was indicated by: * *P* < 0.05, ** *P* < 0.01, *** *P* < 0.001 and **** *P* < 0.0001.

To assess the time-kill effect over time, a 24 h course of AB5075 co-cultured with *Lcb. rhamnosus* CRL2244 with or without addition of fresh medium was assessed and CFU/ml were recorded at different time points (Fig. 2). AB5075 cells were combined with *Lcb. rhamnosus* CRL 2244 in a 1:1 ratio of cultures with OD 600 nm = 0.1. In the absence of fresh medium, cell death of AB5075 was observed at short cell–cell contact time of 4 h (Fig. 2A). In contrast, when fresh BHI medium was added, CFU decreased was observed after six hours with a total cell death after 24 h of incubation (Fig. 2B).

### In co-culture conditions, *Lcb. rhamnosus* CRL 2244 does not induce changes in antibiotic susceptibility

Susceptibility to antibiotics of AB5075 and A118 co-cultured with *Lcb. rhamnosus* CRL 2244 during 4 h with addition on fresh medium was evaluated by the disc diffusion and gradient diffusion. The antibiotic susceptibility profile of A118 and AB5075 strain in the presence of CRL 2244 was similarly to the unexposed strains in the condition tested (data not shown).

### *Lcb. rhamnosus* CRL 2244 induces morphological changes on *A. baumannii* cell

**Figure 3:**
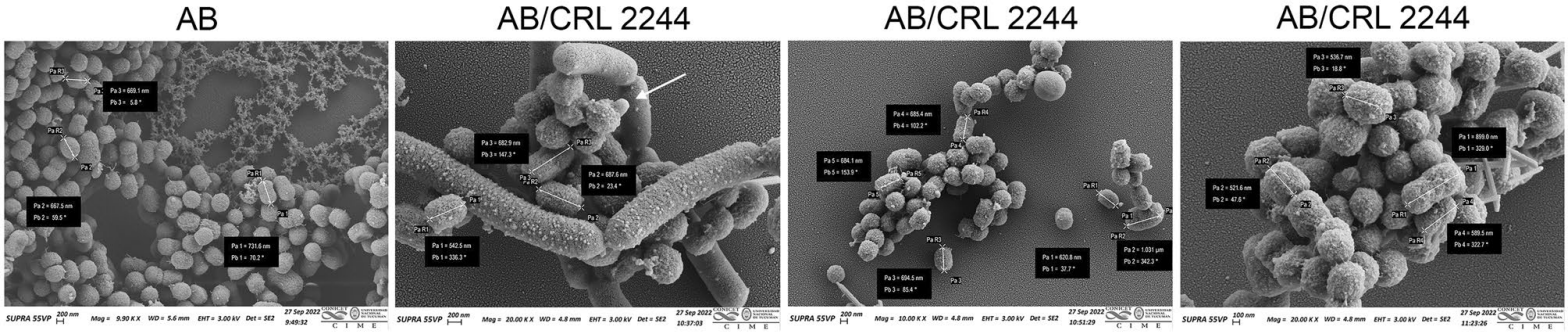
Scanning electron microscopy of *A. baumannii* cells in the presence of *Lcb. rhamnosus* CRL 2244. *A. baumannii* cells cultures were used as control. Micrographs were captured at a magnification of ×10.000 or ×20.000. Bars, 200 nm. White arrow points to a representative outer-membrane vesicle (OMV).

SEM analysis was performed to study if *A. baumannii* experience changes at the morphological level in the presence of *Lcb. rhamnosus* CRL 2244. Microscopic images show that *A. baumannii* cells in the absence of *Lcb. rhamnosus* CRL 2244 (control) are coco-bacillary in shape, uniform in size at approximately 600–700 nm in length, have a rugged surface, and are homogeneously distributed and immersed in an extracellular matrix or biofilm (Fig. 3). When *A. baumannii* was exposed to *Lcb. rhamnosus* CRL 2244, changes in bacterial surface, cell size, and distribution were observed. While the surface remains rugged, the formation of nanotubes and outer membrane vesicles (OMVs) significantly increased compared to control cells grown in the absence of *Lcb. rhamnosus* CRL 2244 (Fig. 3). The size of the cells is variable, some preserving their shape while others show increase in length (sizes of approximately 1000 nm) (Fig. 3). In addition, it is observed that *A. baumannii* cells are distributed forming multicellular three-dimensional conglomerates around *Lcb. rhamnosus* CRL 2244 chains (Fig. 3). Changes in the morphology of *Lcb. rhamnosus* CRL2244 when interacting with A118 were not observed.

### *Lcb. rhamnosus* CRL 2244 induces changes at the transcriptional level of *A. baumannii* in co-culture condition

To investigate the transcriptional response of *A. baumannii* when exposed to *Lcb. rhamnosus* CRL 2244, RNA-seq analysis and quantitative RT-PCR (qRT-PCR) were performed.

RNA-seq transcriptomic analysis of *A. baumannii* AB5075 exposed to *Lcb. rhamnosus* CRL 2244, revealed 386 differentially expressed genes (DEGs) using an adjusted *P* value < 0.05 and a fold-change cutoff of log_2_ > 1. These DEGs represent 10.19% of the total genes in the AB5075 reference genome and encompass a wide range of functional categories. Among the DEGs, 223 were up-regulated and 163 were down-regulated. Notably, these DEGs include genes associated with various important functions, such as iron-uptake, antibiotic resistance, metabolism, cell wall synthesis, virulence, transcriptional regulators, efflux pumps, and motility, among others (Table S2).

**Figure 4:**
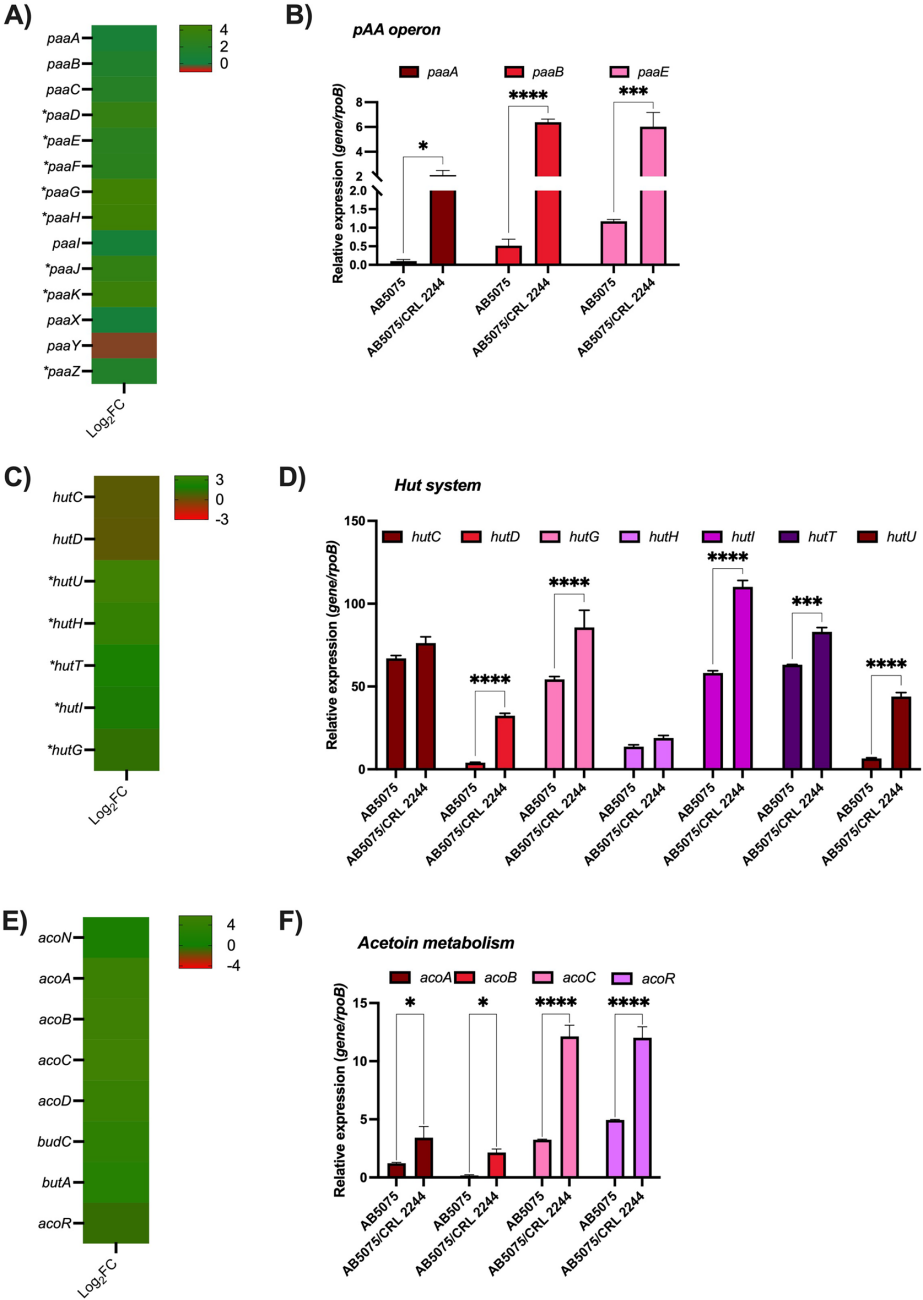
AB5075 transcriptional results of representative metabolic pathways affected by *Lcb. rhamnosus* CRL 2244. (**A**) Heatmap outlying the differential genes expression of genes involved in the phenylacetic acid catabolic pathway. Asterisks represent a *P* value of < 0.05. (**B**) qRT-PCR of *paaA, paaB, and paaE* of AB5075 grew on BHI or in co-culture with *Lcb. rhamnosus* CRL 2244. (**C**) Heatmap outlying the differential genes expression of Hut system codifying genes involved in histidine metabolism. Asterisks represent a *P* value of < 0.05. (**D**) qRT-PCR of *hutC, hutD, hutG, hutH, hutI, hutT,* and *hutU* of AB5057 grew on BHI or in co-culture with *Lcb. rhamnosus* CRL 2244. (**E**) Heatmap representing the differential genes expression genes involved in acetoin metabolism. Asterisks represent a *P* value of < 0.05. (**F**) qRT-PCR of *acoA, acoB, acoC, and acoR* of AB5057 grew on BHI or in co-culture with *Lcb. rhamnosus* CRL 2244. For qRT-PCR assays three independent samples were used. Statistical significance (*P* < 0.05) was determined by two-way ANOVA followed by Tukey’s multiple-comparison test, one asterisks: *P* < 0.05; two asterisks: *P* < 0.01, and three asterisks: *P* < 0.001.

Particularly, there is an increase in the expression of genes involved in metabolic processes, such as the catabolism of organic compounds, amino acids, fatty acids, and carbohydrates (Table S2, Figs. 4, and S1). Genes known to have an impact on the pathobiology of *A. baumannii* and having a role on virulence, immune evasion, infection, and antibiotic resistance^23^, such as the* paa* operon, are differentially expressed in co-culture condition (Fig. 4A and B). PAA catabolism involves the production of succinyl-CoA and acetyl-CoA, which then participate in the tricarboxylic acid (TCA) cycle; there is differential expression of genes encoding different lyase, ligase, transferase and oxido-reductase enzymes associated with these pathways (Table S2 and Fig. 4A and B). In addition, genes of the Hut system (*hutG, hutU, hutH)* involved in histidine catabolism using carbon and nitrogen as source were also found to be up-regulated (Fig. 4C and D). This system has been identified as important for *A. baumannii* infection^24^. Other genes found to be up-regulated were those involved in acetoin/butanediol catabolism (*acoA*, *acoB*, *acoC*, *acoD*, *acoN*, and *acoR*), benzoate metabolism (*benA*, *benB*, *benC*, *benD*, *benK*, and *benP2*) and genes associated with alcohol metabolism (Fig. 4E and F, Table S2 and Fig. S1).

Regarding changes in the expression of genes involved in fatty acid metabolism an increase in the expression of 34 genes was observed in the presence of *Lcb. rhamnosus* CRL 2244. *Lip*A, which is essential for the utilization of long-chain fatty acids, colonization, and persistence in *A. baumannii* infection, was among the observed DEGs (Table S2 and Fig. S1).

**Figure 5:**
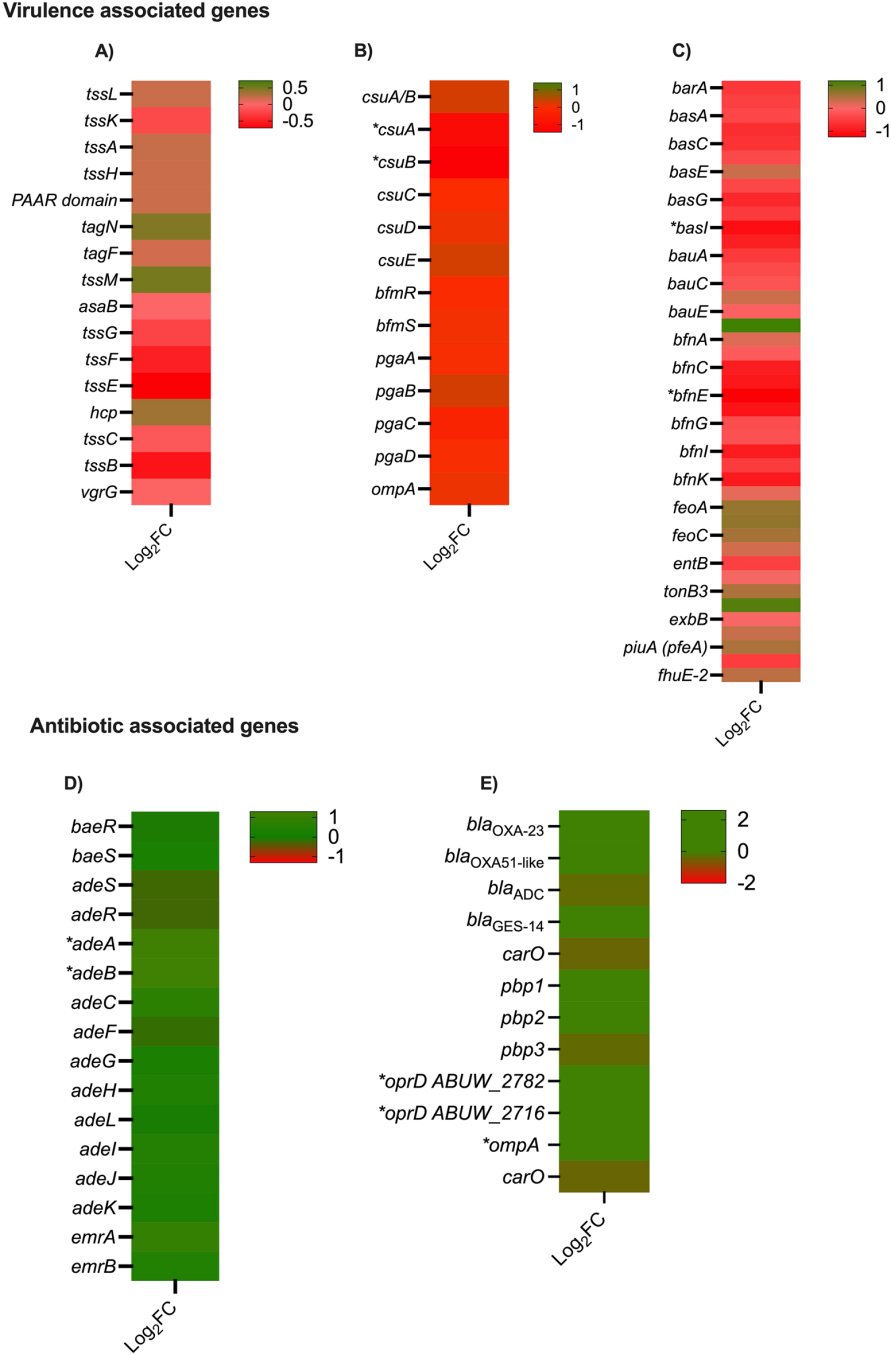
Heatmap representing the differential genes expression of genes involved in virulence and antibiotic resistance. Asterisks represent a *P* value of < 0.05. (**A**) Type 6 secretion systems, (**B**) Biofilm, (**C**) Iron, (**D**) Efflux pumps, and (**E**) antibiotic resistance.

Down-regulation was observed in the expression of genes related to secretion systems, biofilm, and iron metabolism, while up-regulation was seen for antibiotic resistance associated genes (Fig. 5A–E and Table S2). Overnight cultures of AB5075, *Lcb. rhamnosus* CRL 2244 and AB5075 combined with *Lcb. rhamnosus* CRL 2244 for 24 h were used to assess biofilm formation. A statistically significant decrease in the biofilm production was observed when AB5075 was co-cultured with *Lcb. rhamnosus* CRL 2244 (Fig. S2). An increase in the expression of genes related to efflux pumps and antibiotic resistance was observed (Fig. 5D–E). Selected genes were evaluated by qRT-PCR assays, which revealed no statistically significant changes or displayed results that were both in agreement or disagreement with RNA-seq Data (Fig. S3). Lastly, genes encoding LrgB and CidA/LgrA proteins were found to be significantly over-expressed (Table S2). These proteins affect membrane proton motive force and induce programmed cell death in bacteria^25^. In addition, these genes have been identified to be involved in pyruvate uptake system in *Streptococcus mutans* and its role in the connection between cell death and key metabolic pathways^26^.

### Inhibitory effect of *Lcb. rhamnosus* CRL 2244 on a CRAB strain in an environment with other commensal bacteria

The antagonistic activity of *Lcb. rhamnosus* CRL 2244 on AB5075 was evaluated in a complex medium of microorganisms, such human fecal material. For this purpose, commercially obtained human fecal material from a healthy donor was used. *Lcb. rhamnosus* CRL 2244 also affected the viability of AB5075 in the presence of a commensal microbiota. A 35% decrease in AB5075 viability was observed in co-culture with *Lcb. rhamnosus* CRL 2244 relative to control AB5075 (1.3 × 10^8^ *vs* 2.0 × 10^8^ CFU/mL). However, no changes in the phenotypes of surviving AB5075 cells (motility and antibiotic susceptibility profile) were observed. Furthermore, *Lcb. rhamnosus* CRL 2244 reduced the total microbial load of the human fecal material by up to 50% (2.0 × 10^8^ *vs* 4.0 × 10^8^ CFU/mL human fecal control) and was able to inhibit the *E. coli* population present in the human fecal sample (data no shown).

## Discussion

LAB have a long history of safe use in food, where they impart desired technological and nutritional properties to fermented products. In addition, certain species exert beneficial effects on the health of the host. The antagonistic or antimicrobial effect of LAB against pathogens has been well documented in the literature^5,9^. However, studies on the antimicrobial activity of LAB and/or its extracellular products against *A. baumannii* are currently limited^7,9,27^. In the present work we observed a strong inhibitory activity of the LAB *Lcb. rhamnosus* CRL 2244 on susceptible and carbapenem-resistant *A. baumannii* strains. *Lcb. rhamnosus* CRL 2244 exerts a killing effect in *A. baumannii* cells in both exponential and stationary phase. Nevertheless, the supernatant derived from a 24 h cultivation of *Lcb. rhamnosus* CRL 2244 did not show antimicrobial activity within the conditions assessed. It remains plausible that the active metabolite(s)'s concentration was low in the tested milieu. However, the possibility that metabolite(s) released by *Lcb. rhamnosus* CRL 2244 into the environment could exert an effect requires further study.

In addition, the morphology of *A. baumannii* cells displayed alterations when exposed to *Lcb. rhamnosus* CRL 2244. These changes included variations in size, distribution, and the quantity of OMVs released by *A. baumannii*, indicating a potential defensive response, and suggesting that *A. baumannii* cells may be experiencing stress. Ladha et al.^7^, have shown that a pure compound produced by *Lactiplantibacillus plantarum* LJR13 produced pores on the surface of *Listeria monocytogenes*, *S. aureus*, and *A. baumannii* affecting the bacterial surface^7^. Previous studies have demonstrated that exposure of *A. baumannii* cells to lactic acid and acetic acid produced by *Lcb. rhamnosus* can lead to the disruption or disintegration of the *A. baumannii* cell envelope. Additionally, these authors observed the emergence of filament-like structures, which can serve as sites for the excessive leakage of vital cytoplasmic contents^27^.

Through our transcriptomic analysis of *A. baumannii* cell co-culture with *Lcb. rhamnosus* CRL 2244, we observed that the expression of various genes is significantly altered. Interestingly, the majority of differentially expressed genes (DEGs) are associated with metabolic pathways, indicating that *A. baumannii* is actively responding to the stress imposed by the LAB to enhance its survival strategies. The organic acid phenylacetate (PAA) catabolic pathway, which plays a crucial role in various biological processes, is among the metabolic pathways that have been found to be altered. The catabolism of PAA has been associated with immune evasion, colonization, persistence, biofilm formation, oxidative stress, and antibiotic resistance^28–31^. Interestingly, the expression of genes within the *paa* operon is influenced by different environmental conditions, including exposure to human pleural fluid, mucin, and antibiotics, highlighting how *A. baumannii* employs diverse strategies to overcome environmental stress. The increase expression of the *paa* operon has been linked with the repression of the CSU pili, leading to decrease biofilm formation^28^. Our RNA-seq data and biofilm formation assays agree with this study. In our study, we observed the up-regulation of the genes belonging to the Hut system, which is recognized for enabling the utilization of histidine during infection. This finding further reinforces the metabolic adaptability of *A. baumannii*, which is crucial for its survival within the host environment. The metabolism of fatty acids is strongly increased in the presence of the LAB bacteria. Previous reports have shown that host fatty acids have an antimicrobial property during infection^32–34^. Other authors showed that *A. baumannii* β-oxidation may metabolize the toxic fatty acids allowing *A. baumannii* to resist during infection^32–34^. It has also been reported that this species can use fatty acids as energy sources or substrate for membrane biosynthesis^35^. Rodman et al.^36^ observed that when *A. baumannii* is exposed to human pleural fluid or human proteins, such as HSA, there is an increase in the expression of genes associated with fatty acids metabolism^36^. The comprehensive analysis of the transcriptomic data highlights that in the presence of *Lcb. rhamnosus* CRL 2244, *A. baumannii* exhibits a remarkable and versatile metabolic adaptability as a direct response to the stress imposed by the LAB. This adaptability indicates that *A. baumannii* possesses the capability to dynamically adjust its metabolic pathways to respond to the challenges presented by the presence of *Lcb. rhamnosus* CRL 2244. Such adaptability is likely crucial for *A. baumannii's* survival and persistence in the face of environmental stressors.

Another notable finding in the transcriptional response of *A. baumannii* is the significant over-expression of the *cidAllrgA* and *lrgB* genes. These genes have been implicated in programmed cell death and have been previously associated with cellular responses during overflow metabolism^37^. Based on our data, we suggest that the presence of *Lcb. rhamnosus* CRL 2244 can induce stress and potential cellular death in *A. baumannii*, likely due to the heightened metabolic activity observed under these conditions. This observation aligns with the documented phenomenon of *A. baumannii* experiencing cell death after 24 h in the presence of *Lcb. rhamnosus* CRL 2244.

Previous works have shown that probiotics LAB exert their antimicrobial activity by releasing metabolite(s), such as organic compounds or tetramic acids like reutericycline^38^, or by forming multicellular co- aggregations, among others. It has been suggested that cellular co-aggregation, as we have observed by SEM, would allow the colonization of tissues by beneficial lactic acid bacteria, thereby disrupting the pathogen’s adherence^39^. Considering previous published works and the data gathered in this manuscript, we can suggest that when *A. baumannii* is exposed to *Lcb. rhamnosus* CRL 2244, a significant change at the metabolic level can potentially lead to cell death. Future studies will focus on the isolation, characterization, and biological evaluation of *Lcb. rhamnosus* CRL 2244 metabolie(s) to identify specific mechanisms of action that could lead to new therapeutics. Based on previous literature, one potential venue to use the *Lcb. rhamnosus* CRL 2244 or its active compound(s) can be topical administration to treat wound infections^40–42^.

The results presented in this study showed that *Lcb. rhamnosus* CRL 2244 exhibit anti-*A. baumannii* effect. Furthermore, the observed ability of the LAB to induce a broad transcriptomic response in *A. baumannii* highlights the complex interactions between these organisms. This work provides a foundation for future investigations aimed at elucidating the underlying mechanisms involved and the potential of *Lcb. rhamnosus* CRL 2244 to be used to develop novel therapeutic strategies for combating *A. baumannii* infections.

## Materials and methods

### Bacterial strains and culture conditions

Ten lactic acid bacteria (LAB) strains were used to evaluate their antimicrobial activity against *A. baumannii*. The strains included were the model probiotic strain *Lacticaseibacillus rhamnosus* ATCC 53103; and strains with technological and/or functional properties of interest^43–48^, belonging to the CERELA culture collection (CRL) or to the laboratory's collection as *Latilactobacillus curvatus* CRL705, *Limosilactobacillus mucosae* CRL573, *Lactobacillus acidophilus* CRL641, *Fructobacillus tropaeolis* CRL2034, *Limosilactobacillus reuteri* CRL1101, *Companilactobacillus farciminis* CRL748, *Lacticaseibacillus rhamnosus* CRL75, CRL 2244 and Principia (Table S1). The strains were subcultured at the optimum temperature for each one twice in Man, Rogosa and Sharpe (MRS) broth (Oxoid, Basingstoke, Hampshire, United Kingdom) before experimental use.

Two *A. baumannii* model strains, the A118 antibiotic-susceptible strain^49^ and the CRAB AB5075 (*bla*_OXA-23_ and *bla*_OXA-51_,^50^), were used in this work. Also, four additional CRAB clinical strains belonging to different clonal complexes and harboring different types of carbapenemases as AMA16 (*bla*_NDM-1_ and *bla*_PER-7_,^51^), AB0057 (*bla*_TEM-1_, *bla*_OXA-23_, *bla*_ADC_,^52^), ABUH702 (*ISAba*1/OXA-66^53^), and AYE (*bla*_VEB-1_, *bla*_OXA-69_^54^) were tested. The strains were grown at 37 °C in BHI broth (Oxoid, Basingstoke, Hampshire, United Kingdom) with agitation.

### Antagonistic or inhibitory activity of lactic acid bacteria on *A. baumannii* strains

The antagonistic or inhibitory activity of LAB against the different *A. baumannii* indicator strains was determined by soft agar overlay method^5^. Briefly, spots (10^5^ CFU/spot) of culture (C), supernatant (S) and pellet (P) (S and P obtained by centrifuging the culture at 10,000 rpm for 5 min) of different LAB were inoculated onto MRS agar plates, using a culture in MRS broth (grown at 37° or 30 °C for 24 h), and allowed to dry for 30 min. They were then covered with Muller-Hinton (MH) soft agar (0.8% agar) premixed with 10^8^ CFU of *A. baumannii* indicator strains and incubated at 37 °C for 24 h. The diameter of the *A. baumannii* growth inhibition halo (IHD) was measured and interpreted according to Shokryazdan et al.^55^: the IHD > 20 mm (Strong); 20–10 mm (intermediate); and < 10 mm (weak). The width of the clear zone or "R" value was also determined according to the formula R = Inhibition diameter—Spot diameter divided by two^5^; and will be interpreted "no inhibition capacity" when R < 2 mm, "low inhibition" with "R" values of 2–5 mm, and "high inhibition capacity" with "R" values > 6 mm^56,57^. AU/ml (arbitrary units per ml) was calculated as the IHD × 1000 divided by the μl seed^5^. The assays were performed in triplicate.

### Killing assay

The viability of *A. baumannii* AB5075 and A118 in co-culture with *Lcb. rhamnosus* CRL 2244 was assessed by a) combining ON cultures of both cells (1:1 ratio of cultures with OD 600 nm = 0.1) with no addition of fresh medium and b) combining 1:1 ratio of cultures with OD 600 nm with addition of fresh BHI medium, incubated at 37 °C for 24 h. *A. baumannii* and CRL 2244 independent cultures were used as controls. CFU/mL were determined at different incubation times by decimal dilutions and plated on CLDE medium (Sigma-Aldrich, St. Louis, MO, USA) with and without the addition of erythromycin (2 μg/mL) to kill *Lcb. rhamnosus* CRL 2244. Statistical significance (*P* < 0.05) was determined by two-way ANOVA followed by Tukey’s multiple comparison test, using GraphPad Prism (GraphPad Software, San Diego, CA, USA).

### Antibiotic susceptibly assays

Susceptibility to antibiotics of AB5075 co-cultured with CRL 2244 during 4 h was evaluated by the disc diffusion and gradient diffusion (minimum inhibitory concentration) following the procedures recommended by the Clinical and Laboratory Standards Institute^58^. Commercial antimicrobial discs (Liofilchem S.r.l., Italy) of 30 μg of cefepime (FEP), 30 μg of ceftazidime (CAZ), 10 μg of imipenem (IMP), 10 μg of meropenem (MER), 30 μg cefiderocol (FDC), amikacin (AK), 5 μg of ciprofloxacin (CIP) were used and the plates were incubated at 37 °C for 18 h. MICs to cefiderocol (FDC) and tetracycline (TE) were performed by MTS (Liofilchem S.r.l., Italy) following manufacturer’s recommendations. Assays were performed in triplicate.

### Scanning electronic microscopy

From overnight cultures of A118 in BHI broth (incubated at 37 °C with shaking) and CRL2244 in MRS broth (incubated at 37 °C), 500 μl of each were combined (co-culture) and incubated at 37 °C without shaking for 1 h 30 min. Controls included individual cultures of both cells. The samples were then centrifuged at 500 rpm for 2 min. To the collected pellet, 1 mL of Karnovsky fixative pH 7.2 (2.66% paraformaldehyde, 0.1 M sodium phosphate buffer and 1.66% glutaraldehyde) was added and homogenized. Then, 100 μL were placed on the surface of a 10 mm diameter glass coverslip, allowed to dry for one hour and dehydrated using a battery of alcohol solutions of increasing gradation 30, 50, 70, 90, 100° and acetone for 10 min each. After acetone, critical point drying was performed in Denton vacuum equipment model DCP-1. The glasses were mounted on an aluminum support (stub) and adhered by means of a double-sided conductive carbon tape. They were then coated with gold in a JEOL model JFC-1100 ion sputter. The electron microscopic observation was performed with the ZeissSupra 55VP Scanning Electron Microscope (Germany) belonging to the Centro Integral de Microscopia Electronica (CIME-CONICET- Universidad Nacional de Tucumán).

### RNA extraction sequencing and RNA-seq data analysis

Overnight cultures of *A. baumannii* AB5075 and CRL 2244 with OD adjusted to 600 nm at 4.0 were centrifuged and resuspended in 3 mL of BHI broth and incubated for 90 min at 37 °C. Total RNA extractions were performed in three biological replicates for each condition (AB5075 combined with CRL 2244 and AB5075) using Direct-zol RNA Kit (Zymo Research). After checking the absence of DNA contamination, Novogene Corporation (CA) was outsourced to perform mRNA-seq analysis, which included rRNA depletion, library preparation following the protocols of the NEBNext Ultra II Directional RNA Library Prep Kit for Illumina (New England Biolabs) and Illumina NovaSeq 6000 paired-end 150 bp sequencing. The RNA-seq reads (GEO accession GSE236782) corresponding to *A. baumannii* AB5075 exposed to CRL 2244 were analyzed as follows. First, Trimmomatic v0.39 was used to trim low-quality bases at the ends of the reads to a minimum length of 100 bp and remove Illumina adaptor sequences. Then, FastQC (www.bioinformatics.babraham.ac.uk/projects/fastqc/) was used to assess the quality of the reads before and after trimming. The RNA-seq reads were aligned to whole genome sequence of AB5075 using Burrows-Wheeler Alignment software (BWA). FeatureCounts was used to calculate the read counts per gene, and DEseq2 was employed to perform differential expression analysis. Features exhibiting FDR < 0.05 and log2fold change > 1 were considered statistically significant.

### Quantitative reverse transcription polymerase chain reaction (qRT-PCR)

The extracted and DNase-treated RNA was utilized to generate complementary DNA (cDNA) following the manufacturer's protocol provided with the iScriptTM Reverse Transcription Supermix for qPCR (Bio-Rad, Hercules, CA, USA). cDNA concentrations were adjusted to 50 ng/µL and qPCR was conducted using the qPCRBIO SyGreen Blue Mix Lo-ROX following manufacturer’s protocol (PCR Biosystems, Wayne, PA, USA). At least three biological replicates of cDNA were used in triplets and were run using the CFX96 TouchTM Real-Time PCR Detection System (Bio-Rad, Hercules, CA, USA). Transcriptional levels of each sample were normalized to the transcriptional level of *rpoB*. The relative quantification of gene expression was performed using the comparative threshold method 2^-ΔΔCt^. The ratios obtained after normalization were expressed as fold changes compared to cDNA samples isolated from bacteria cultures individually, and asterisks were used to indicate statistically significant differences, as determined by ANOVA followed by Tukey's multiple comparison test (*P* < 0.05), using GraphPad Prism (GraphPad Software, San Diego, CA, USA).

### Biofilm formation assay

Overnight cultures of AB5075, CRL 2244, and AB5075 combined with CRL 2244 were incubated in BHI at 37 °C for 24 h. The optical density at 600 nm (OD_600_) was adjusted to 0.9–1.1 and 100 μl placed in a 96-well polystyrene microtiter plate and incubated at 37 °C for 24 h without shaking. The next day, the OD_600_ (ODG) was measured, using a microplate reader, to determine the total biomass. The wells were emptied, washed three times with 1X phosphate buffered saline (PBS) and stained with 1% crystal violet (CV) for 15 min. Excess CV was removed by washing three more times with 1X PBS and the biofilm associated with CV was solubilized in ethanol acetate (80:20) for 30 min. OD_580_ (ODB) was measured and the ratio of biofilm to total biomass (ODB/ODG) was determined. Experiments were performed in triplicate and statistical significance (*P* < 0.05) was determined by two-way ANOVA followed by Tukey's multiple comparison test using GraphPad Prism (GraphPad software, San Diego, CA, USA).

### Human fecal material and interaction assays

A stock solution of commercially obtained healthy donor human fecal material (Innovative Research, USA, certified vendor approved by ISO, FDA, USDA, and EPA) was prepared by dissolving 5 g in 50 mL of sterile ultrapure water. From active cultures of CRL 2244 grown in MRS broth and AB5075 in BHI broth and adjusted to OD600 = 0.1, two hundred μl of each cell was combined with the addition of 900 μl of fecal matter and 900 μl of BHI broth (final volume 2 mL). AB5075 cells were used as controls. After incubation at 37 °C for 24 h, the viability of AB5075 and changes in phenotyping were determined:

- Cell viability (CFU/mL): using differential media such as CLDE (with and without the addition of erythromycin 2 μg/mL), Levin; and selective media such as CHROMagar™ *Acinetobacter* (Chromoagar, Paris, France).
- Motility assay in soft agar (0.5% agarose): LB plates were prepared with 0.5% agarose were used. From the colonies obtained in the cell viability assay, colonies were picked and seeded on the surface of the motility plate and incubated at 37 °C for 24 h. Growth diameter was measured and classified as non-motile (< 5 mm), moderately motile (5–20 mm) or highly motile (> 20 mm). Experiments were performed in triplicate.
- Antibiotic susceptibility assays: From the colonies obtained in the cell viability test, susceptibility was evaluated to cefepime (FEP), ceftazidime (CAZ), imipenem (IMP), meropenem (MER), cefiderocol (FDC), amikacin (AK), gentamicin (GN), ciprofloxacin (CIP), trimethoprim/sulfamethoxazole (TMS) and tetracycline (TE) using the disc diffusion technique and determination of cefiderocol (FDC) and tetracycline (TE) minimum inhibitory concentration, following the procedures recommended by CLSI (2020).

### Supplementary Information

Supplementary Information 1.

Supplementary Information 2.

Supplementary Information 3.

Supplementary Information 4.

Supplementary Information 5.

Supplementary Information 6.

## Data Availability

The datasets generated and analyzed during the current study are available in the Gene Expression Omnibus (GEO) repository, (GEO accession No GSE236782 or token number glcdkswudzkvzuv).
