# Supplementary figures and images for: Antimicrobial activity of the Lacticaseibacillus rhamnosus CRL 2244 and its impact on the phenotypic and transcriptional responses in carbapenem resistant Acinetobacter baumannii
Source: Sci Rep. 2023 Aug 31;13:14323. doi: 10.1038/s41598-023-41334-8 (PMC10471627; doi:10.1038/s41598-023-41334-8)

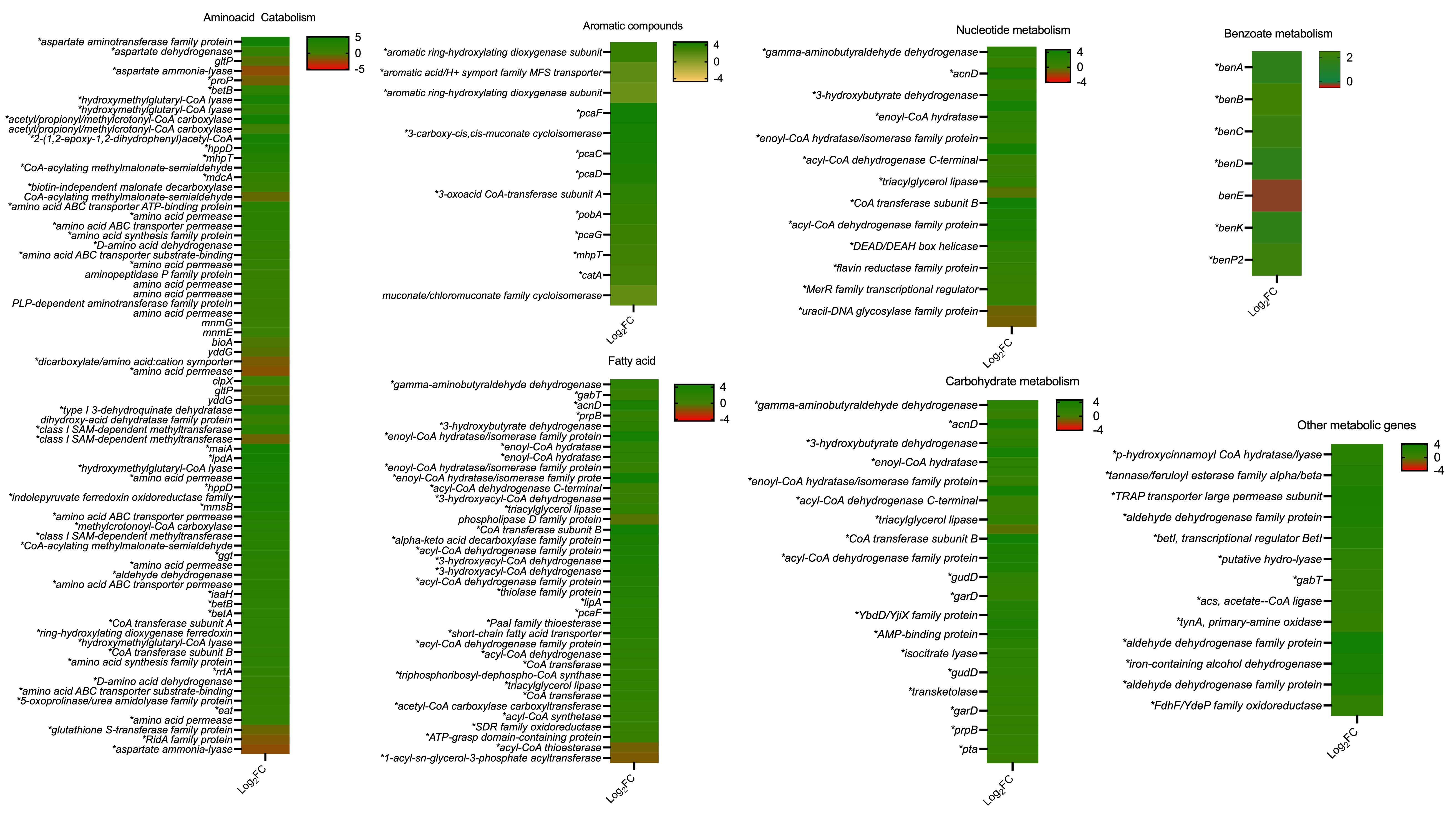

Supplement: Supplementary file 1 — Supplementary Information 1. [file 41598_2023_41334_MOESM1_ESM.jpg]

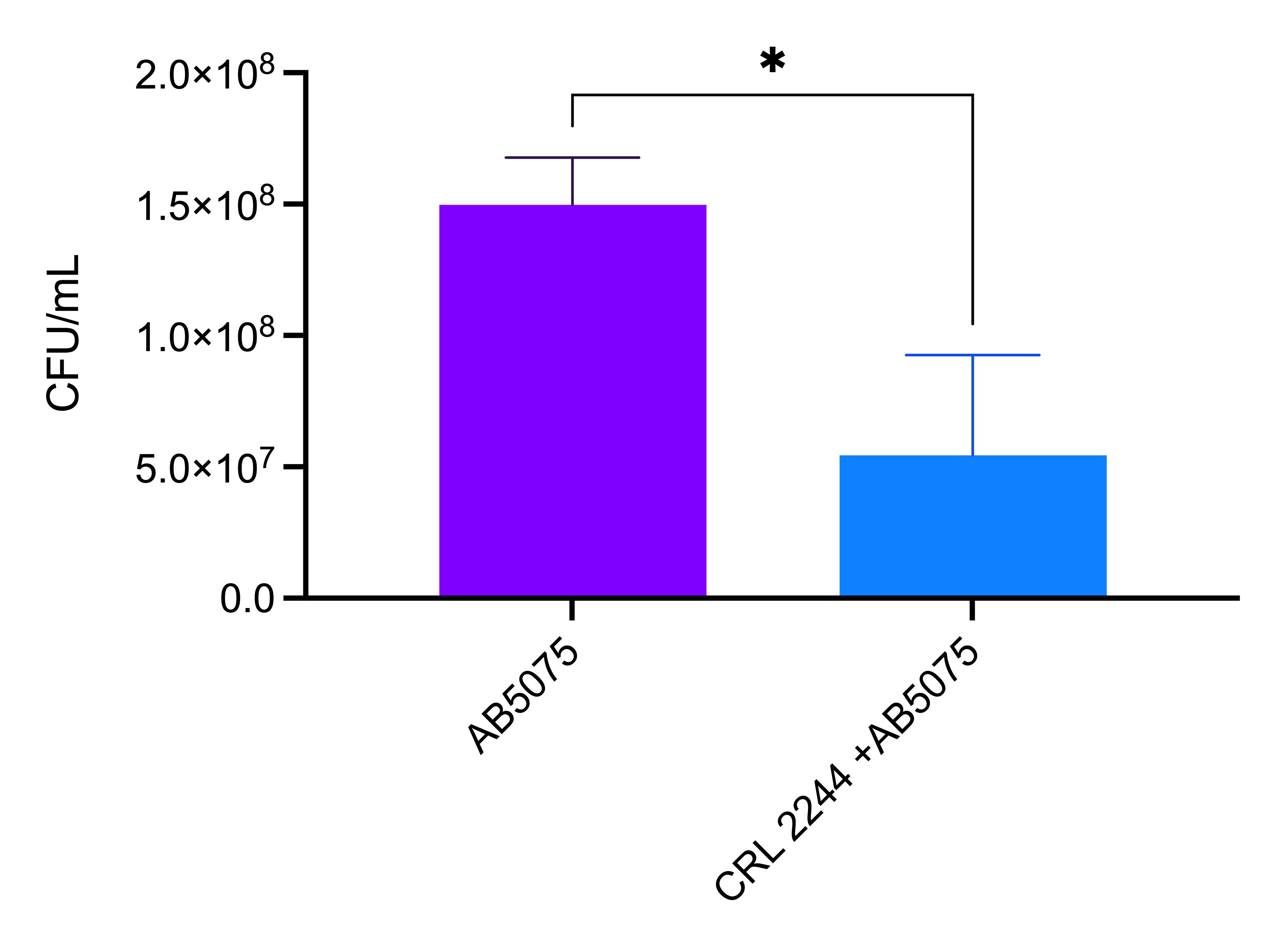

Supplement: Supplementary file 2 — Supplementary Information 2. [file 41598_2023_41334_MOESM2_ESM.jpg]

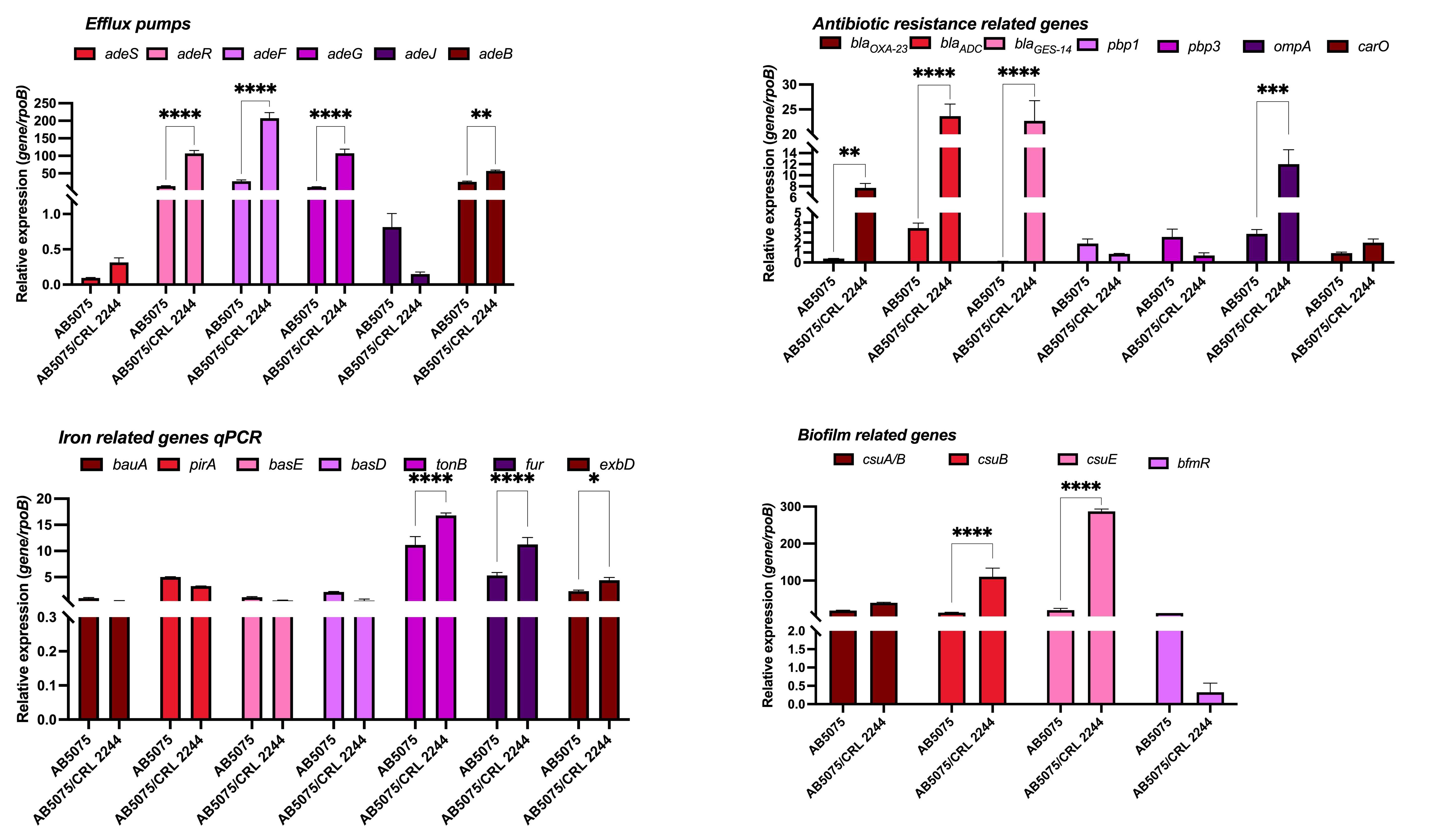

Supplement: Supplementary file 3 — Supplementary Information 3. [file 41598_2023_41334_MOESM3_ESM.jpg]
