# Supplementary material for: Antimicrobial activity of the Lacticaseibacillus rhamnosus CRL 2244 and its impact on the phenotypic and transcriptional responses in carbapenem resistant Acinetobacter baumannii
Source: Sci Rep. 2023 Aug 31;13:14323. doi: 10.1038/s41598-023-41334-8 (PMC10471627; doi:10.1038/s41598-023-41334-8)
Supplement: Supplementary file 4 — Supplementary Information 4. [file 41598_2023_41334_MOESM4_ESM.docx]

**Legends of Supplementary Material.**

**Table S1**. Lactic acid bacteria used in the antimicrobial activity studies.

**Table S2.** List of AB5075 ORFs. Green log2-fold change >2, red log2-fold change <2, yellow p-adjusted value >0.05.

**Figure S1.** Heat map of other key metabolic pathways that were differentially expressed in *A. baumannii* strain AB5075 upon exposure to *Lcb. rhamnosus* CRL 2244. Asterisks represent a *P-value* of <0.05.

**Figure S2.** Phenotypic analysis of biofilm formation. Biofilm assays performed with the AB5075 strain grown in BHI co-culture. The mean ± SD is informed of three independent experiments. Statistical significance (*P <* 0.05) was determined by ANOVA followed by Tukey’s multiple comparison test, one asterisks: *P* *<* 0.05; two asterisks: *P* < 0.01 and three asterisks: *P* < 0.001.

**Figure S3.** qRT-PCR of genes associated with efflux pumps, antibiotic resistance, iron and biofilm. qRT-PCR assays were performed in three independent experiments and the statistical significance (*P* < 0.05) was determined by two-way ANOVA followed by Tukey’s multiple-comparison test, one asterisks: *P* < 0.05; two asterisks:
