# Supplementary material for: Antimicrobial activity of the Lacticaseibacillus rhamnosus CRL 2244 and its impact on the phenotypic and transcriptional responses in carbapenem resistant Acinetobacter baumannii
Source: Sci Rep. 2023 Aug 31;13:14323. doi: 10.1038/s41598-023-41334-8 (PMC10471627; doi:10.1038/s41598-023-41334-8)
Supplement: Supplementary file 5 — Supplementary Information 5. [file 41598_2023_41334_MOESM5_ESM.docx]

**Table S1.** Lactic acid bacteria used in the antimicrobial activity studies

| **Species** | **Strains** | **Source** | **Properties** | **Culture conditions** |
| --- | --- | --- | --- | --- |
| *Latilactobacillus curvatus* | ^a^CRL705 | Handmade salami | Bacteriocin producer  (Vignolo *et al*, 1996) | MRS broth/30^◦^C 16 h |
| *Limosilactobacullus mucosae* | CRL573 | Child feces | Mannitol producer  (Rodriguez *et al*, 2012) | MRS broth/37^◦^C 16 h |
| *Lactobacillus acidophilus* | CRL641 | Fermented milk product | Antimicrobial producer  (Segli *et al*, 2021) | MRS broth/37^◦^C 16 h |
| *Fructobacillus tropaeoli*s | CRL2034 | Fig | Mannitol producer  (Ruiz Rodriguez *et al*, 2017) | MRS + fructose (2% p/v) broth/30^◦^C 16 h |
| *Limosilactobacillus reuteri* | CRL1101 | Silage | Reduce inflammatory and oxidative damage during endotoxic shock  (Juarez *et al*, 2013) | MRS broth/37^◦^C 16 h |
| *Companilactobacillus farciminis* | CR748 | Artisanal Argentinean sausage | Not described | MRS broth/37^◦^C 16 h |
| *Lacticaseibacillus rhamnosus* | ATCC 53103 | American Type Culture Collection | Inflammatory bowel disease prevention (Yan y Polk, 2012) | MRS broth/37^◦^C 16 h |
|  | CRL75 | Unknow | Immunomodulatory  (LeBlanc *et al*, 2011) | MRS broth/37^◦^C 16 h |
|  | CRL 2244 | Wastewater | Not described | MRS broth/37^◦^C 16 h |
|  | Principia | Commercial yogurt | Probiotic effect | MRS broth/37^◦^C 16 h |
|  |  |  |  |  |

^a^CRL, CERELA culture collection. ^b^MRS: Man, Rogosa and Sharpe medium.
